# Ecologies of Public Trust: The NHS COVID-19 Contact Tracing App

**DOI:** 10.1007/s11673-021-10127-x

**Published:** 2021-10-05

**Authors:** Gabrielle Samuel, Frederica Lucivero, Stephanie Johnson, Heilien Diedericks

**Affiliations:** 1 Department of Global Health and Social Medicine, King’s College London, Bush House, Strand, London, UK; 2 Wellcome Centre for Human Genetics, University of Oxford, Oxford, OX3 7BN UK; 3 Ethox Centre and Wellcome Centre for Ethics and Humanities, Oxford University, Oxford, UK

**Keywords:** Contact tracing app, Digital health, Ethics, Trust, Trustworthiness

## Abstract

In April 2020, close to the start of the first U.K. COVID-19 lockdown, the U.K. government announced the development of a COVID-19 contact tracing app, which was later trialled on the U.K. island, the Isle of Wight, in May/June 2020. United Kingdom surveys found general support for the development of such an app, which seemed strongly influenced by public trust. Institutions developing the app were called upon to fulfil the commitment to public trust by acting with trustworthiness. Such calls presuppose that public trust associated with the app can emerge if the conditions for trustworthiness are met and that public trust is simplistic, i.e., linearly the sum of each member of the publics’ individual – U.K. government trust relationship. Drawing on a synthesis of the trust literature and fifteen interviews with members of the public trialling the app on the Isle of Wight, this paper aims to explore what trust mechanisms and relationships are at play when thinking about public trust in the context of the U.K. COVID-19 app. We argue that public trust is a complex social phenomenon and not linearly correlated with institutional trustworthiness. As such, attention needs to widen from calls for trustworthy infrastructures as a way to build public trust, to a deeper understanding of those doing the trusting; in particular, what or whom do people place their trust in (or not) when considering whether using the app and why. An understanding of this will help when trying to secure public trust during the implementation of necessary public health measures.

**Supplementary Information:**

The online version contains supplementary material available at 10.1007/s11673-021-10127-x.

## Introduction

To combat the COVID-19 public health emergency, in 2020 policymakers around the globe increasingly invested in digital health technologies to support the “isolate, test and trace” approach of containing virus spread. These technologies included mobile “contact tracing” applications (apps), which could trace individuals likely to have come into contact with those who had reported symptoms or tested positive for the virus and request that they self-isolate (WHO 2020). Contact tracing apps were heralded as offering potential public health benefits for better pandemic management, including the ability to forecast outbreaks, improve healthcare efficiency, and communicate with the public (Budd et al. 2020; Morley et al. 2020).

Alongside this, the development of contact tracing apps raised heated debate about possible intrusions of privacy and concerns about increased surveillance (Sharon 2020; Wienroth et al. 2020), including concerns about who would have access to the data collected via these apps and the re-purposing of the data for other uses (Anderson 2020). Concerns were also raised that app usage could reinforce digital divides, exacerbate inequalities, and/or discriminate against certain groups (AdaLovelace Institute 2020a, b; Gasser et al. 2020; Kahn et al. 2020; Morley et al. 2020; NHSX app Ethics Advisory Board 2020).^1^

On April 12, 2020, close to the start of the first U.K. lockdown, the U.K. government announced the development of a National Health Service (NHS) contact tracing app. This app was trialled on the Isle of Wight during May and June 2020. Following the trial on the Isle of Wight, this app was withdrawn, and the NHS developed a new app, which was launched for general population use in September 2020.

At the time of the original app’s development, U.K. surveys found general support for the development of such an app, though reportedly a significant number felt anxious about the government’s ability to protect personal data, as well as the perceived potential for government surveillance post-pandemic (Abeler et al. 2020; Duffy 2020). Support, or lack thereof, was reflected and seemed to be strongly influenced by public trust in the decisions made around the app and the system of which it was part (AdaLovelace Institute 2020a, b). These findings correlated with scholars’ more general calls for the need to foreground public trust during the development of contact-tracing apps (Bengio et al. 2020; Kahn et al. 2020; Ranisch et al. 2020). Institutions developing the app were called upon by scholars and commentators alike to fulfil the commitment to public trust by acting with trustworthiness: scholars demanded attention to transparent and open governance processes, accountability, clarifications around key ethical issues, independent assessment of the technology, and considerations of vulnerable groups (NHSX app Ethics Advisory Board 2020; Parker et al. 2020).

Such calls for trustworthiness seemingly presuppose that public trust is simplistic, i.e., linearly the sum of each member of the publics’ individual – U.K. government trust relationship and can emerge if the conditions for trustworthiness are met (openness, transparency, etc.). However, a growing literature has explored the nature of public trust in relation to the health sector, health systems, and practices (see for example Aitken et al. 2016). This literature argues that public trust is better described as an emergent social property depending on several factors that cannot be understood by referring to individual persons or institutions alone. Rather, public trust is situated between the individual, the health system, the state and other societal institutions such as the media and social media (Gille et al. 2017). Extensive work on social trust in psychology (Siegrist et al. 2000; You 2012; Brandt et al. 2015), social epidemiology (Subramanian et al. 2002; Feng et al. 2016), sociology (Welch et al. 2005; Dinesen and Sønderskov 2015), and political science (Herreros and Criado 2008; Rothstein and Eek 2009), suggests similar findings. Given these various definitions of trust, it is relevant to ask what trust mechanisms and relationships were at play (for example, linear or complex) when thinking about public^2^ trust in the context of the U.K. NHS COVID-19 app. This was the question that guided our research, and its importance relates to the need for policymakers and public health officials to understand how public trust is formed. This will allow them to better ensure public trust is secured when necessary public health measures need to be implemented.

We conducted fifteen interviews with members of the Isle of Wight public during the app trialling period in May 2020. Our aim was to explore what trust mechanisms and relationships played a role in constructing interviewees’ views about the app. To do this, we synthesized an understanding of how different disciplines have addressed the concept of trust in the broader literature. This has so far not been conducted. From this, we distilled which concept would be most useful and relevant to our analysis. We drew on the notion of “ecologies of trust” to guide the analysis (Steedman et al. 2020). We present our findings and discuss them. Before this, we present our synthesis of the literature and the concept of “ecologies of trust.”

## A Synthesis of the Literature on Trust

### Interpersonal and Impersonal Trust

The dominant paradigm used to explore the various dimensions of trust is based upon accounts of *interpersonal trust*—trust between two individuals known to each other (McLeod 2002) and *impersonal trust*, concerning trust in strangers and social systems (Delhey and Newton 2003). Interpersonal trust conceptualizes and measures trust as a property of the individual and includes both psychological perspectives that theorize trust as a core personality characteristic (Glanville and Paxton 2007) and sociological perspectives that view trust as related to characteristics like income, class, and race (Alesina and Ferrara 2002). Impersonal accounts of trust view trust as an emergent property of societies/communities (Delhey and Newton 2003). This includes both thin/generalized trust in unknown others [often from different social backgrounds or social systems with which we have frequent but fleeting contact (Delhey et al. 2011)], and thick/particularized trust extended to people who “are like us” (religion, race etc.) regardless of whether we know them or not.^3^

### Mediating “Trust-Mechanisms”

Both interpersonal and impersonal trust are formed through processes rooted in different “trust-mechanisms.” These include (1) “calculus-based” trust grounded in a rational gamble that the other will act in your interest because they have an interest in fulfilling the trust; (2)”knowledge-based” trust is rooted in previous experiences; and/or (3) “identification-based” trust related to emotional ties, shared values, and altruism (Lewicki and Bunker 1996; Hardin 2002).^4^ These forms of trust can exist in parallel, for example, trust in the NHS may involve knowledge-based experiences of interpersonal trust based on interactions with a clinician but could also involve an impersonal trust in the social system of the NHS, based on the shared values of social welfare (Gilson 2003). As such, the manifestation of trust is complex and unlikely reducible to a single component, such as rationality or affective ties. Rather, trust has been argued to be viewed as a relational process of both inter- and impersonal trust relationships, forming a “complex ecology of trust,” including the individual’s “experience, perception, understanding and feelings as they relate to organisations, services, people and practices” (Steedman et al. 2020, p. 825).

### Public Trust

Public trust is a type of impersonal trust. The notion of public trust has been increasingly and extensively mobilized as a necessary requirement for public health endeavours, as well as the legitimacy of new technologies that involve the collection, storage, use, or re-use of data. Public health scholars have made efforts to define the concept of *public* trust. This, they say, can be separated from individual trust (based on a person or group’s characteristics; also called intrinsic or interpersonal trust) and institutional trust (based on the institution in which an individual is embedded; also called extrinsic trust and a type of impersonal trust) (Critchley and Nicol 2011), both of which have consistently been identified as important factors in ensuring public willingness in health research and/or acceptance of healthcare contexts (Lucassen, Montgomery, and Parker 2017; Samuel and Farsides 2018; Burgess and O’Doherty 2019; Tindana et al. 2019). Public trust, rather, is perceived as an emergent property of societies that needs to be understood as “a distinct *social phenomenon* that co-exists with individual trust” and as something that originates in the public sphere (Gille et al. 2017). Public trust is not only influenced by the trustworthiness of a recipient institution of the trust but also by individuals’ perceptions of this trustworthiness, as well as its media image of individuals’ experiences and other social sectors and industries that have recognizable impacts on the system, such as the national/multinational private sector and advocates (Gille et al. 2017).

### Social Trust

The description of *public* trust has significant similarities to *social trust*, which refers to how much a person trusts other people in general (Newton 2001), although there is little overlap between the literatures. A social trust perspective implicitly assumes that some population or macro-level factors influence individuals independently of that individual’s particular characteristics (Jen et al. 2010). Central to this theory of trust is relationality since both societal and individual levels of trust have an interactive effect (Welch et al. 2005). For example, trust relations formed through interpersonal interactions, such as a patient’s relationship with her clinician, are crucial for trust in the medical institution more broadly, acting as access points reaffirming the institution’s trustworthiness (Gilson 2003). *Social* trust, contrasts with *political* trust—more used in political science—which refers to trust in the political institution or public institutions (Levi and Stoker 2000).

### The Complex Ecology of Trust

As the above highlights, public trust is predominantly viewed as a relational process of different types of trusting mechanisms and relationships, forming a “complex ecology of trust.” This ecology describes the different ways that “multiple factors engender, maintain or undermine trust … including experience, perception, understanding and feelings as they relate to organisations, services, people and practices” (Steedman et al. 2020, p. 825). This theorization of trust is advantageous as it can bring together the different theories and notions of trust described above, including public trust, in a usable way, by allowing the acknowledgement of the complexity of trust and its varying components. We drew on this theorization to explore the complex interplay (ecology) of trust mechanisms and relationships associated with beliefs about the app. If, as we argue, public trust is complex rather than linear, then an understanding of these complex mechanisms and relationships is crucial for those aiming to secure public trust for the implementation of necessary public health measures. To identify trust mechanisms and relationships in our data, we focused on which types of trusting and trust relationships were present. We matched these forms of trusting to those presented in our synthesis above.

## Methods

### Interviews

Interviews were conducted with residents of the Isle of Wight, since this was the only location where the first NHS COVID-19 app—which is the focus of this study—was trialled. Interviews were part of a broader project exploring interviewees’ views and, where relevant, experiences associated with this NHS COVID-19 app. As such, interviews did not focus on trust. Interviews asked about interviewees’ information-sourcing relating to the app, their decision to download the technology (or not), experiences of using the app, perceptions about the benefits and harms, and views more generally about the app, and contact tracing more broadly.

### Data Collection

Fifteen phone/online interviews were conducted by GS/HD in May 2020. Inclusion criteria included individuals who were over 18 years old and at the time residing on the Isle of Wight. Recruitment was online via a local Isle of Wight Facebook page advert (with moderator permission), a newsletter, an advertising site (*Wightbay*), and snowballing. Interviews lasted between sixteen and sixty-seven minutes, with n = 10 between thirty and fifty minutes. Table 1 highlights interviewee demographics. All interviewees had previous experience of downloading mobile phone apps. Thirteen interviewees had downloaded the NHS app. One interviewee had received an alert to isolate from the app (interviewee 10), and one interviewee had used the app to request a test (interviewee 3).

**Table 1:** Interviewee demographics

	App Downloaded	App Not Downloaded
Gender		
Female	8	0
Male	5	2
Age (years)		
18–39	2	1
40−69	9	1
70 plus	2	0
Education		
16 years	2	1
18 years	2	0
College*	2	0
Undergraduate	3	0
Postgraduate	4	1
Employment Status		
Employed	5	2
Unemployed	1	0
Furloughed	3	0
Retired	4	0

^*^College is non-University education post-school from 16 years of age

### Analysis

All authors read the transcripts and inductively coded two interviews independently. Coding was shared and discussed, and from this a coding structure was generated and applied to all interviews by GS and HD (HD coded transcripts; GS checked for consistency with her own coding). Additional codes were added during coding, and these additional codes were shared between GS and HD in an iterative process of constant communication and reflection on the data. Codes were discussed with all authors and themes developed. The project codebook (Appendix 1 in Supplementary Information) is included for reference. Themes were then mapped onto the various trust mechanisms and definitions identified in the literature.

Ethics approval was received from King’s College London research ethics office: MRA-19/20–19251.

### Limitations

The small sample size of self-selected interviewees recruited online is always a limitation of interview studies because it only allows the capture of the views of those who have seen the advert as well as those who wish to be interviewed. Furthermore, we could only interview members of the public who resided on the Isle of Wight, since these were the only individuals who were trialling the app at the time. There is the possibility that the island has its own culture and community, meaning that the views of our interviewees may not reflect national views more generally. The aim was to continue the analysis at a national level to explore how these findings compared to the views of wider U.K. members of the public, however, at the time the trial was halted, there was political uncertainty about when the new app would be released, if at all, and so a decision was made to end data collection at the end of June 2020.

## Findings

### Good Communication is Essential But Not Vital for Accepting the App

Several interviewees (n = 3) perceived the government had provided appropriate and clear information about the app. Most, however, disagreed. Interviewees described little access to information about the organizations responsible for the app’s development, as well as to information about how exactly the app worked; “when I downloaded the app, I couldn’t find any information … telling me what it was going to do, it was just going to alert me if I was in proximity, but it didn’t say when, how long” (interviewee 7). Interviewee 3 had questions around inputting data into the app, as well as about the security risks of leaving the Bluetooth mechanism on their phone permanently. They explained; “there isn’t anywhere to ask those questions…I did email them…but the answer I got back was worse than useless.” Some interviewees explained how app-associated information was unhelpfully presented. Others mentioned how poor information provided by government was doing little to allay people’s worries about the data, how it would be handled, who would have access to it, and for what purposes; “it was just never explained very well about the app, and I think it should have been done far, far better, everybody was frightened of privacy, nobody seemed to understand how it worked” (interviewee 15).

Despite most interviewees raising these concerns, as we show later, most interviewees were still accepting of the technology, and all but two interviewees (interviewees 4 and 11) downloaded the app. Reasons for not downloading the app included technological limitations (interviewee 11 had an older model phone) and critical beliefs about the app and the U.K. government more broadly ((interviewee 4); see below).

This suggested a more complex relationship between the U.K. government (failing to) provide open and transparent information about the app (something called for by scholars as an aspect of trustworthiness) and views on the technology. In fact, interviewees highlighted different trust relationships that were entangled with the information provided about the app. For instance, some interviewees who expressed frustration with information gaps about the app were concerned this could increase the trust people placed in misinformation from other sources. They explained that without relevant information about how the app would function and how data would be handled, used and stored, a “space” is created “for people to fill it with their imagination …” (interviewee 6) and to breed misinformation and further distrust; “it takes one person to say something like that [negative remark about the app] and then everybody who doesn’t really know latches on to it” (interviewee 6). In this way, interviewees perceived that it was a rational gamble to trust the U.K. government when so little information was given (impersonal calculative distrust) and that the U.K. government did not share their values (identification-based distrust). As such, interviewees suggested that an impersonal calculative distrust towards the information supplied by the U.K. government, coupled with identification-based distrust led some to trust other sources of information. These other sources of information might have been more clearly presented to them (so they can take the rational gamble); might have been from sources who share values/beliefs with the truster (identification-based); and/or might have come from sources whom the trustor might have a history of “good experiences” of trusting (knowledge-based). In short, some interviewees perceived the lack of U.K. government information created a lacuna to be potentially filled with other information that is trusted based on all three trust-mechanisms.

Other trust relationships beyond the information provided by the U.K. government also seemed to influence interviewees’ views on the app. Below we explore these, beginning with interviewees’ pre-existing views (and *political* trust in) the U.K. government.

### Political Trust is Important but Not Essential for App Acceptance

Interviewees shaped at least some of their perceptions about the app by the impersonal *political* trust they placed (or not) in the U.K. government, as well as in the government’s handling of the COVID-19 pandemic. On the one hand, for most interviewees who tended to speak more supportively of the app, also tended to speak in more supportive terms about the government, or at least their comments during the interviews seemed to be more understanding of the government’s decisions during the pandemic and more generally. In contrast, for a minority of interviewees who were exceptionally critical of the government’s decisions during the pandemic, they were concomitantly worried about the government’s handling of the app development. This revolved around two key issues—whether the government had the required competency to deliver the technology and their perceptions about the government’s underlying motivations and future intentions for the app’s implementation in society. Such views were most clearly highlighted by interviewees 1 (who downloaded the app) and 4 (who did not download the app). Interviewee 1 was particularly worried about the government’s ability to develop an app that was both workable and useful. Explaining their initial thoughts on the app, they drew on knowledge-based trust to remark; “it’s probably not going to be a great app in the world because it’s sorted out by the UK Government and with everything else that's gone wrong …” These concerns seemed to play out for this interviewee after they downloaded the app; they spoke about a range of issues they were having with relation to installing the app on their phone and interpreted these issues in line with their views of the U.K. government who “have not got it right again*.*” This interviewee’s concerns chimes with a knowledge-based *distrust*: the interviewee has repeated past experiences/perceptions of the U.K. Government reneging on promises made to the public with little or no negative consequences. As such, the interviewee is sceptical about trusting in the realization of new Government promises, such as a functioning track and trace app.

Interviewee 4, who was generally concerned about how much control the government had over society and had repeatedly pointed to the notion of “Big Brother,” was anxious that the app was being developed by government to “control” society; “the government…are looking at different ways to implement control, and it’s like using apps and technology like this to implement the next stage in control.” Other interviewees who did not express this concern directly, also spoke of “others” on the Island who were worried that the app was being used as a way for the government to “track” them (interviewee 6). These interviewees described a broader suspicious U.K. culture that is generally untrusting and “anti-government”; “we are very suspicious in this country, we don’t trust anything…we are always fighting against the government” (interviewee 5). Interviewee 4’s concerns may well bely an identification-based *mistrust*: they do not share the values or societal norms espoused by the current government, such as the value of state health security via surveillance.

Pre-existing *political* trust in the government therefore played an important role in shaping beliefs about the app. A remark by interviewee 13 nicely illustrates this point; “there were some attempts … to … describe how the data [for the app] would be used. But … for me personally it comes down to … how much trust I have in them [the U.K. government] to actually tell the truth.” However, as we saw earlier, there was not a simple relationship between political (dis)trust and interviewees being unsupportive of the app, indeed interviewee 1, who was very critical of the government downloaded the app. Furthermore, while other interviewees acknowledged that whether they deemed the government to be competent to deliver the app, to have good intentions when implementing the app (not “big brother” intentions) and/or being truthful was paramount, they also described several other influencing factors. These included the information provided (described above), interpersonal trust in individuals “selling” the app in stories they heard from their peers, in the media, and in social media, all of which played a role in formulating their opinions about the app. Below we focus on two of these to exemplify this: the influence of individuals (government representatives, expert communicators selling the app) and social networks and social media.

### Trust in Government Representatives and Expert Communicators

Interviewees’ narratives highlighted the importance of who was communicating about the app when considering trust. Several interviewees described how perhaps politicians were not the best placed individuals to “sell” the app to the public; interviewee 6 suggested; “I think it needed a technical person not a politician [to explain the app,] because they don’t have a lot of credibility”; interviewee 14 noted “I think it’s all about how it’s sold.” Expertise was therefore seen as an important aspect in ensuring that trust is placed in those communicating to the public about the app. It also illustrates how *interpersonal* trust relationships to politicians are tied to pre-existing *impersonal political* trust. The influence of these pre-existing beliefs on perceptions was made most apparent when several interviewees spoke about an incident in which Bob Seely—a local Member of the U.K. Parliament and app advocate—was caught attending a barbecue during lockdown. Interviewee 13, who generally spoke critically about the U.K. government’s handling of the pandemic, reflected on their frustration that government officials did not adhere to lockdown rules. As such, this interviewee spoke about the difficulties they had in *knowledge-based* trusting “what the government are saying” about the app;

Dominic Cummings’ lockdown break, and the same with our local MP, he was seen to be not following the lockdown rules … people in charge need to be leading by example and if they are not leading by example then it feels like we are being told to do one thing while other people in positions of power are not following that same advice. So, it makes it very difficult to trust the things they are saying in other respects. (interviewee 13)

In contrast, interviewee 15, a Conservative Party member who openly expressed their shared values with the Party,^5^ justified this event in line with their own views, and remained supportive about the app’s development, speaking to an *identification-based* trust;

I have the T-shirt “where the Isle of Wight leads the rest follow.” I think that Bob Seelly probably had quite a bit to do with that, I like Bob, obviously I’m biased … The barbecue rubbish and all that, if everybody is whiter than white … and what have you. But I believe him when he said … he thought he was just going for a chat … (interviewee 15)

These findings move us away from a simple relationship between accepting the app, the U.K. government acting with trustworthiness, and *political* trust. Rather, an interplay of pre-existing beliefs and different factors in “trusting” was apparent, including the behaviour of the wider U.K. government, as well as who was communicating about the app (interpersonal trust). As such, trust is not reducible to information provided (or not provided) but reflects the interplay between the identification of shared values, perceptions of “being let down before” and issues of perceived fairness regarding rule-following, as the Bob Seelly incident illustrates.

### Social Networks and Social Media

Interviewees’ conversations and “gossip” with others (interviewee 9) via “the Isle of Wight grapevine” (interviewee 3) played an important role in interviewees’ sourcing information about the app; “we all talked about it at work … me and my husband spoke about it and friends … everyone was talking” (interviewee 5). A number of go-to local Facebook sites were also used by residents to discuss the app, and all interviewees spoke about using these sites.^6^ Interviewees therefore used social networks and social media to fill perceived information gaps from the government. How much trust interviewees placed in the information they sourced from social networks (*interpersonal* trust; offline, friends, acquaintances) and social media [*impersonal*, placed in the social media system, or *interpersonal*, placed in specific social media users mediated online (Keymolen 2016)] seemed to be entwined with their pre-existing political trust in the U.K. government (as described above) and/or their pre-existing trust in the app. Indeed, as we show below, pre-existing trust in the app was itself related to interviewees’ more general trust in science and technology. In other words, these pre-existing beliefs appeared to influence which information they aligned with.

In particular, those interviewees less accepting of the app and/or the U.K. government, aligned themselves with negative online comments about the technology that reinforced their view. Interviewee 1 from above, who was sceptical about the government’s competence to develop the app to start with, particularly reflected on these issues when reading online about the app’s technological limitations. Highlighting a form of *identification*-based trust with social media users who were perceived to share their values regarding the app, this interviewee explained that by discussing these issues online, they had decided to uninstall the app; “with the information that I got … online, and people that I spoke to, I decided to uninstall the app.” Interviewee 4, from above, was similarly influenced by online comments reinforcing their expressed distrust in the government—this time with relation to concerns that the app was acting as a tool for societal control.

In contrast, those interviewees who were seemingly more supportive of the U.K. government and/or more accepting of the app, were more sceptical of online comments; “I read some of the negativity and the positivity on … Facebook … and basically ignored all the naysayers” (interviewee 3); “it’s more rumour, it's more crap that’s out in the ‘twatesphere”’ (interviewee 15). Interestingly, when not talking specifically about the app, these same interviewees provided examples of *trusting* social media as a source of information, suggesting that the influence of social media in affecting these interviewees’ beliefs about the app was linked to whether the stories and comments they read aligned with their pre-existing beliefs about the app and/or the U.K. government. These findings, together with those above, create a complex picture of the interplay between different trust relationships and pre-existing beliefs as they related to acceptance of the app. Indeed, accepting the app became a filtering and balancing process of pre-existing ideas about the U.K. Government, with existing knowledge, as well as information from social networks and social media sites, and individuals “selling” the information.

### Future Orientated Trust

Overall, the sense of looming threat and a desperation to control the virus, coupled with a commonly held understanding that research and technology is intrinsically good, and a sense that the app had a health benefit, all seemed to play an important role in shaping interviewees’ perceptions of whether to use the technology. All but two interviewees had downloaded and used the app, regardless of their expressed views on the U.K. government and reported trust in the app to function. So, although participants spoke directly about their (dis)trust in the government, in practice they all placed their trust in the app to offer potential health benefits.

Nearly all interviewees were hopeful that the app would bring an end to the pandemic by helping control the spread of the virus. Interviewees described the concept of an app as “a good idea” (interviewee 12), “a very useful tool” (interviewee 13), and as a “technological solution” (interviewee 11) to helping contain the pandemic given the limited resources available for manual contact tracing. For these and some other interviewees, this hope was sometimes mixed with misconceptions about the potential for the app to deliver individual benefit, in addition to population health benefit. Indeed, the potential for health benefit seemed closely connected with the threat COVID-19 posed; that is, the health benefit was synonymous with risk management: “I downloaded it … because I wanted to keep safe” (interviewee 15); “I have taken my [app] off, now I feel less safe … [..] … more vulnerable … [..]… I haven't got any of that safeguard at the minute” (interviewee 14). In this way, the threat of COVID-19 seemed to mitigate how interviewees enacted trust—the risk COVID-19 posed, and therefore the benefit or protection the app ostensibly promised, trumped any concerns they had about the app and/or the U.K. government.

The extent of perceptions about the app as providing benefit also seemed connected to interviewees’ understanding of the app, as well as their pre-existing beliefs about science as an intrinsically good endeavour. Some interviewees, who tended to place a lot of trust in the intrinsic value of research and science, equally placed a lot of hope that the app would bring about a population health benefit. In other words, these interviewees trusted the promise associated with the app to deliver these benefits. Other interviewees, who tended to have a more pragmatic outlook on what science could deliver to society in general, were less inclined to assume that the app could help control the pandemic but were pragmatically in strong support of trialling the technology because of the *potential* health value; “I think if it does actually help, if people are traced and it does work, that way then yes by all means use it” (interviewee 10). Interviewee 9, for example, reflected they would “give it a go.” These interviewees perceived little harm could come from trialling the app and could articulate few concerns relating to its use; “I thought, well there is no harm in it at all, so go for it” (interviewee 8); “I don't really see many downsides to having it …” (interviewee 11). Even those interviewees whose support for the app was shaped by their negative view of government, or by the technology’s reliability issues and limitations, and/or concerns about potential infringements on privacy (via “Big Brother” narratives), perceived a value in trialling and/or using the app in the hope of controlling the spread of the virus. Only two interviewees—the two that had particular concerns with the government—were apprehensive about the role of the app in containing the pandemic, viewing the promotion of the app as more about playing on people’s fears of the virus to ensure they downloaded the app, either as a way of “fear-mongering” to elicit control on the population (interviewee 4), or to give them a sense that they were doing something, rather than nothing; “it seems [the app] is more of a psychology thing to deal with people’s mental frame of mind rather than a function that it does this, it does that” (interviewee 1).

Despite these criticisms, interviewee 1, who explicitly described their fear of the virus, still downloaded the app. Interviewee 4—who had little concern or fear about the virus (and who did not trust the general public concern about the virus), did not download the app.

## Discussion

While other studies have empirically explored participants’ perspectives about COVID-19 contact tracing apps, as well as how such apps have been discussed and debated in the public domain (for example, see Williams et al. 2020; Amann, Sleigh, and Vayenna 2021), this is the first study that we know of that has *empirically* explored the concept of public trust as it relates to such apps. Analysing our data by drawing on the conceptualization of “ecologies of trust” allowed us to identify a range of trust mechanisms and types that were associated with interviewees’ views of the original U.K. COVID-19 NHS app trialled on the Isle of Wight. These included political trust, calculus-based, knowledge-based, and identification-based trust; interpersonal and impersonal trust; and political trust. Our findings also highlighted a range of inter-connected, pre-existing beliefs and trust relationships related to the app, including with the U.K. government, individuals, private corporations, social networks, social media, the media, science more generally, the capabilities of the app, the threat of the virus (fear), and the promise of the technology to help control the pandemic. Our findings therefore show that public trust in relation to this particular app did not simply imply that individual people trust the app to work or they trust the government. Rather, trust in the app needed to be understood in the context of the ecology of trust relationships (Steedman et al. 2020) that interplay into the complex social phenomenon of public trust (Gille et al. 2017). Below, we explore this ecology that emerged in the findings and discuss the implications.

First, our findings are consistent with work in other contexts that have shown trust in government is an important determinant of citizens’ compliance with public health policies, especially in times of crisis (Blair et al. 2017; Vinck et al. 2019). Our interviewees’ pre-existing beliefs, or trust, in the U.K. government influenced their views on the app. Particularly for those who were less supportive of the government’s development of the technology, their engagement with others’ experiences and wider perceptions of the app and of government, had an influence on their perceived trust (Gille et al. 2017).

However, second, our data goes beyond *political* trust relationships to show that developing trust in the app was a process of checking and filtering information from a variety of sources, events, and expertise. Pre-existing ideas around government, government actors, and science and technology influence this process, as well as a perceived vulnerability to the virus and hope for a solution to the pandemic. One aspect of this process relates to the interpersonal aspect of trust. Despite some literature arguing that an institution’s character cannot be reduced to the character of its members (Kerasidou 2017), our interviews showed that the perceived virtue of government representatives played a role in engendering public trust. This aligns with the idea that communication cannot succeed if the speaker is perceived to have violated certain ethical or rule-following norms—e.g. lying (O’Neill 2002; Manson and O’Neill 2007). A speaker cannot “inform” a distrustful audience. References to Bob Seeley and Dominic Cummings in our interviews show that the behaviour of individual actors had an undermining impact that the required institutional trust, and this should not be under-emphasized. Moreover, the expertise (or not) of such individuals played a role in engendering public trust, which is another factor that needs to be reflected upon. Here, the trust relationship is grounded on the acceptance of an asymmetry of expertise/knowledge: I trust the clinician’s diagnostic skills because she is a medical expert, but I do not necessarily trust my clinician’s cooking skills because this is not an epistemic advantage that I have reason to acknowledge (unless I know she was a chef before becoming a doctor). In this sense, the U.K. government’s support of the app was seemingly problematic because they were not perceived to have the skills to know why and how the app worked. While the U.K. government pitched most decisions in their pandemic response strategy as guided by the science (Alwan et al. 2020), the endorsement of the app was made by political actors (Matt Hancock, Dido Harding) whose expertise was not necessarily acknowledged and publicly recognized.

Third and finally, another aspect relates to the amount of hope our interviewees had that the app would help contain the virus, and alongside this, the concerns of risk associated with the pandemic (fear for themselves and/or others). This aspect is under-discussed in the trust (interpersonal/impersonal) literature. Hope is a trust relationship, understood in terms of trusting that the app will help contain the virus when there is little other way to do so. In fact, our capacity to hope supports our capacity to trust (McGeer 2008; Sheikh, and Hoeyer 2018). Though trust based on hope needs to be questioned (Smith 2021) because it is based on hope for something that may never be realized, and could therefore be viewed as “misplaced trust,” as a therapeutic misconception, or as “undue influence” (see Sheikh and Hoeyer 2018). In fact, misconceptions can alter perceptions of risk associated with the virus, which can lead to behaviours that are not effective in preventing the spread of disease (Majid et al. 2020). This was evident when some interviewees spoke about their misconceptions about the individual health benefits perceived to be garnered from using the app. The sociology of expectations literature has given much attention to the relationship between promises and hope. This literature describes how promissory hype instils hope in emerging technologies, which is later dashed when the technology fails to deliver on initial promises. Dashed hope gives way to renewed hope as promises are made again, this time about newer technologies (the “hype cycle” [Brown 2003]). Given the sociality of public trust, and its relationship to hope, it is likely that the amount of trust our interviewees placed in the app may in fact mirror promissory hype cycles and it would be interesting for future research to explore this further.

Overall, our data shows that trust is not binary but a spectrum where degrees of trust are given to different actors and institutions (Camporesi et al. 2017). Our sample was small, and our study was limited to only members of the public trialling the first U.K. contact tracing app on the Isle of Wight. However, even from this small analysis, our findings identified the complexity of how public trust is formed and performed. While in different circumstances, the factors contributing to this may be different, we wish to emphasize the complexity inherent to *how* public trust is formed. Trust is not a quality conferred to a person or object but a relational and iterative process of comparing, filtering, and balancing pre-existing ideas, hopes, and fears, and that trust is placed in individuals (on the Isle of Wight this was Bob Seeley), institutions (the U.K. Government), and things (the app).

These findings have critical implications. First, creating public trust requires more than institutions fulfilling the commitment to public trust by acting with trustworthiness—as called for by so much of the ethical literature discussing contact tracing apps, highlighted in the introduction. Such calls ignore the interpersonal and other impersonal trust relationships relevant to members of the public. The need for trustworthiness is, of course, unquestionable. As we have emphasized elsewhere (Samuel and Lucivero 2021; Samuel, Roberts et al. 2021), the U.K. government could have facilitated this with more openness and transparency about the app’s development, which, in turn, could have bolstered its public perception as a trustworthy institution. However, public health scholars’ attention needs to widen from only establishing trustworthy infrastructures to including those doing the trusting as well (Steedman et al. 2020); and in particular, how users frame and respond to risk more generally. User beliefs in science and the representation of expertise are all important considerations. Second, broad policy references to public survey data and “trust” can divert attention away from members of the public’s hopes and concerns, which can end up legitimizing the use of the app without paying adequate attention to the actual reasons people choose to take part in the trial i.e., that they trust in the hope (and promise (*forthcoming*)) of the app to contain the virus (Sheikh and Hoeyer 2018). If/when the COVID-19 pandemic is brought under control, an awareness of these other reasons will be vital in understanding people’s continuing and/or changing views about the app, which in turn will shape the success of such interventions in the future. An awareness of other reasons will also be crucial for future health crises and pandemics. For example, if there comes a point where the virus is less of a public concern but citizens are still advised to use the app, understanding why individuals trusted the app initially may help to explain user behaviour in terms of whether they choose to continue using the app when feeling less threatened or anxious about the health risk.

## Supplementary Information

Below is the link to the electronic supplementary material.

Supplementary file1 (DOCX 16 KB)

## Footnotes

1 Both in the U.K. and internationally, the debates associated with contact tracing appsincluding the form that this technology should take (decentralized vs centralized)were extensive and provoked much media attention.

2 We are aware that the public constitutes a range of “publics.” However, we approach the concept of “public trust” as a single social phenomena that is not the sum of its parts.

3 This latter trust can lead to certain social dangers, such as discrimination against those “not like us” and fanaticism about those that are (e.g. fascism).

4 For example, 1) the patient trusts their doctor because they are aware that there are deterrents in place (such as medical legal action) preventing the doctor from breaking their trust; these deterrents also run counter to the doctor’s best interest; 2) Based on many previous experiences with trustworthy doctors, the patient comes to know that doctors are trustworthy, thereby trusting their current doctor; 3) The patient comes to know the doctor and learns they share key values (e.g. pro-life values) and trusts the doctor on this basis.

5 The Conservative party was in power during the COVID-19 pandemic.

6 Interviewees were recruited via on-line methods, which might explain this.
