# Supplementary material for: Ecologies of Public Trust: The NHS COVID-19 Contact Tracing App
Source: J Bioeth Inq. 2021 Oct 5;18(4):595–608. doi: 10.1007/s11673-021-10127-x (PMC8490841; doi:10.1007/s11673-021-10127-x)
Supplement: Supplementary file 1 — Supplementary file1 (DOCX 16 KB) [file 11673_2021_10127_MOESM1_ESM.docx]

**Appendix 1: Coding structure**

**BENEFITS OF THE APP**

BENEFITS benefits of the app - family

BENEFITS benefits of the app - self

BENEFITS benefits of the app - society and others

BENEFITS lack of benefits - others

BENEFITS lack of benefits – self

BENEFITS societal benefit

**COMPARING**

COMPARING other apps

COMPARING to other countries' strategies

COMPARING to other national emergencies

COMPARING to other viruses/pandemics

**CONCERNS**

CONCERNS - security - no concerns - others

CONCERNS controlling

CONCERNS data uses

CONCERNS people's misuse of the app - spoofing etc including purpose and accidental

CONCERNS privacy - descriptive

CONCERNS privacy - no concerns - others

CONCERNS privacy - no concerns - self

CONCERNS privacy - others

CONCERNS privacy - self

CONCERNS private company/big tech - concerns - others

CONCERNS private company/big tech - concerns - self

CONCERNS private company/big tech - descriptive

CONCERNS private company/big tech - no concerns - others

CONCERNS private company/big tech - no concerns - self

CONCERNS reliability

CONCERNS resources

CONCERNS security - concerns - others

CONCERNS security - concerns - self

CONCERNS security - descriptive

CONCERNS security - no concerns - others

CONCERNS security - no concerns - self

CONCERNS surveillance - concerns - others

CONCERNS surveillance - concerns - self

CONCERNS surveillance - descriptive

CONCERNS surveillance - no concerns - others

CONCERNS surveillance - no concerns - self

CONCERNS use of Bluetooth

conflating issues of contact tracing with issues of app

**DEMOGRAPHICS**

DEMOGRAPHICS not downloaded

DEMOGRAPHICS phone pinged or knows of a phone that did

DEMOGRAPHICS received notification from the app - self or others

DEMOGRAPHICS self-reporting symptoms on the app - self or other

**EXPECTATIONS**

EXPECTATIONS negative

EXPECTATIONS positive

**GOV AUTHORITIES AND THE APP**

GOV referring to the government

GOV referring to the government and the app

**INFO AND COMMUNICATION ABOUT THE APP**

INFO enough/not enough info on participating in trial

INFO evaluating information

INFO hearing stories about the app

INFO importance of information

INFO information sourcing

INFO reading gov guidance about the app

**LIMITATIONS**

LIMITATIONS -- can't take app to work/doesn't take phone everywhere

LIMITATIONS app layout

LIMITATIONS app not enforceable

LIMITATIONS need incentives/gamify app

LIMITATIONS need international app

LIMITATIONS no reminders/push notifications

LIMITATIONS people coming from mainland don't have app

LIMITATIONS/discrimination -- can't afford smart phone/phone too old

**OTHER CODES**

OTHER doing your part/good citizen

OTHER mimicking media

OTHER modernity compared to privacy issues resignation to privacy

OTHER need for UK innovation and drawing on stories as rationale for app etc

OTHER positioning the I of W

OTHER referring to commercial entities not with relation to the app

OTHER referring to other countries

OTHER referring to the healthcare system

OTHER refers to manual tracing

OTHER the importance of individual actors

OTHER app not that important in people's lives - not coming up in conversation

OTHER centralised vs decentralised

OTHER viewing app as passive

OTHER not viewing app as trial

OTHER nothing to hide

OTHER intrinsic value of research

OTHER free code

**PERCEPTIONS OF THE APP**

PERCEPTIONS (not) understanding contact tracing/apps

PERCEPTIONS silver bulleting

PERCEPTIONS views about the app - negative - others

PERCEPTIONS views about the app - negative - self

PERCEPTIONS views about the app - positive

PERCEPTIONS views about the app - positive - others

PERCEPTIONS changing perceptions of the app

PERCEPTIONS GP viewing app as quickest most secure way for testing

PERCEPTIONS need an app given pandemic situation

PERCEPTIONS of other lockdown strategies

PERCEPTIONS of other people’s opinions about the app

PERCEPTIONS of other people's behaviour

PERCEPTIONS OF THE VIRUS

PERCEPTIONS OF VIRUS - uncertainty and threat

PERCEPTIONS putting the app in perspective

**USING APP**

USING compliance with the app - self or others

USING consent

USING experiences of having app on phone

USING Installing the app

USING technical difficulties with the app

USING uninstalling the app
